# Clinical manifestations and prognosis of unexpected uterine sarcoma of uterine fibroids in Tianjin China

**DOI:** 10.1186/s12905-022-02077-2

**Published:** 2022-12-06

**Authors:** Guangyan Cheng, Yuanjing Hu, Yingying Gong

**Affiliations:** 1 Department of Gynecological Oncology, Tianjin Central Hospital of Gynecology Obstetrics, Tianjin Key Laboratory of Human Development and Reproductive Regulation, 156 Nankai Third Road, Nankai District, Tianjin, 300100 People’s Republic of China; 2 Clinical College of Central Gynecology and Obstetrics, Tianjin Medical University, Heping, Tianjin, 300070 People’s Republic of China

**Keywords:** Morcellation, Uterine fibroids, Unexpected uterine sarcoma

## Abstract

**Background:**

Uterine sarcoma is a rare malignancy of women and fewer uterine sarcomas are detected preoperatively. The reported incidence of preoperatively diagnosed uterine sarcoma (PDUS) was 0.07%. This study aims to identify the prevalence of unexpected uterine sarcoma (UUS) after operation for presumed leiomyoma and compare clinical outcomes after primary therapy.

**Methods:**

A retrospective study was performed evaluating all uterine sarcoma diagnosed in Tianjin Central Hospital of Gynecology and Obstetrics between May 2011 and July 2016.We used the χ^2^ and T tests to assess the incidence and clinical features of patients. The Kaplan–Meier method was used to calculate disease-related survival.

**Results:**

The study retrospectively analyzed 6625 patients with uterine fibroids and found 45 UUS patients and 21 patients of PDUS. The incidence of UUS is (45/6625) 0.67%. The incidence of UUS in patients undergoing total hysterectomy was higher undergoing tumor resection (*P* < 0.001); the age of UUS is younger than PDUS (*P* = 0.046); the differences in menopausal status and primary complaints between the two groups are not statistically significant. The PDUS group had more patients with Stage II and III sarcomas than the UUS group (*P* < 0.001); the duration of symptoms in the PDUS group was longer than in the UUS group (*P* = 0.033). The 5-year overall survival (OS) rate of the UUS group (77.7%) is higher than the PDUS group (46.3%) (*P* < 0.001).

**Conclusions:**

The incidence of UUS is low. UUS has a younger age of onset, shorter history of the disease, earlier clinical stage, and better prognosis.

## Background

Uterine sarcoma is a rare malignancy of women, comprising only 1% of all malignancies of the female genital tract and 3–7% of all uterine tumors [1]. Globally, the average age at diagnosis is 55 years [2]. The vast majority of patients have no specific signs. Up to 25% of patients are asymptomatic [3]. The preoperative diagnosis of uterine sarcoma is challenging because of its nonspecific clinical manifestations and the similarity of symptoms to those of uterine fibroids. Additionally, the lack of specific tumor markers and effective diagnostic measures for early-stage disease results in uterine sarcoma being discovered unexpectedly by intraoperative frozen-section examination or postoperative pathological diagnosis in most patients [4]. Some researchers use the term "patients with unexpected uterine sarcoma" for patients who have undergone myomectomy or total hysterectomy for uterine fibroids and have been found by postoperative pathological examination of the operative specimen to have uterine sarcoma [5]. Several studies and systemic reviews have revealed that the rate of unexpected uterine sarcomas among women who undergo surgery for presumed benign leiomyomas ranges from 0.1% (1 in 2,000) to 0.4% (1 in 278) [6, 7]. And another study reported that the incidence of UUS was 0.12% or 1:865 patients. Preoperatively, a malignancy was unexpected in 46% of the cases and expected in 54% [8].

In 2014, FDA issued a recommendation restricting the use of power morcellation, and even in the 2020 update, the use of a power morcellator is contraindicated in principle, and providing sufficient information to patients when using it is necessary (it may affect the prognosis if it is malignant) [9]. More than half of uterine sarcomas are misdiagnosed as uterine fibroids preoperatively and some of them are subjected to inappropriate surgical procedures, particularly the use of morcellators, which can contribute to the spread of sarcomas and severely adversely affect patients' prognosis [10].

The objective of this study was to evaluate the risks of unexpected uterine sarcoma and investigate the clinical manifestations.

## Methods

### Patients and inclusion and exclusion criteria

We retrospectively analyzed 6625 patients with uterine fibroids who had undergone surgery in Tianjin Central Hospital of Gynecology and Obstetrics between May 2011 and July 2016. Our study was conducted by Helsinki Declaration principles. This study was approved by the committee of the Medical Ethics department from the Central Hospital of Gynecology and Obstetrics in Tianjin. Patients with uterine fibroids who had undergone surgery and pathological examination of the surgical specimens revealed uterine sarcoma were included. Patients with incomplete clinical-pathological data, accompanying other systemic malignant diseases (not including metastases from uterine sarcoma), who were diagnosed pathologically as not having sarcoma in other hospitals consulted by our hospital, and who were suspected preoperatively of having sarcoma but subsequently found to not have sarcoma were excluded. By the above inclusion and exclusion criteria, 21 patients with preoperative diagnoses of sarcoma and 45 UUS women were identified. The study cohort comprised these 45 UUS patients and 21 patients of PDUS clinical and surgical datums contemporaneously.

### Diagnostic criteria of PDUS and UUS

Uterine sarcomas are malignant uterine mesenchymal tumors including uterine leiomyosarcoma, endometrial stromal sarcoma, uterine adenosarcoma, and undifferentiated uterine sarcoma [11]. NCCN guidelines classify uterine carcinosarcoma as endometrial cancer. PDUS groups were defined as those with preoperative imaging examination or diagnostic curettage and pathological diagnosis of uterine sarcoma, and postoperative pathological diagnosis of uterine sarcoma. Patients with high suspicion of uterine sarcoma on preoperative imaging were included in the PDUS group. Preoperative pelvic MRI was performed when gynecological ultrasonography showed uterine fibroids with abundant blood flow, ill-defined borders, degeneration of fibroids, and rapid growth of uterine fibroids in a short period of time. Among them, 16 were diagnosed with uterine sarcoma by MRI, and 5 were diagnosed with uterine sarcoma by preoperative curettage. UUS were diagnosed as uterine fibroids before surgery and were diagnosed as uterine sarcomas by intraoperative freezing or postoperative pathology.

### Study parameters

Study parameters were classified into three categories: surgical approaches, clinical features and prognosis. Surgical approaches were included laparoscopic surgery, abdominal surgery, and hysteroscopic surgery. Clinical features were included age and menopausal status*,* clinical staging, patients’ chief complaints and duration of symptoms. The 2018 International Federation of Gynecology and Obstetrics (FIGO) staging system was used for all uterine sarcomas.

### Statistical analysis

SPSS20.0 statistical software was used to analyze the incidence and clinical features of uterine sarcoma. The χ2 test was used for all numeric data and Student’s *t*-test for measurement data. OS was defined as the period from diagnosis to either the day of death or the last visit. Use the Kaplan–Meier method to draw a survival curve, and a log-rank test to compare survival rates. *P* < 0.05 is considered statistically significant.

## Results

### Incidence of UUS and proportion of pathological diagnoses

Forty-five of 6625 (0.67%) patients operated on for uterine fibroids were postoperatively pathologically diagnosed as having uterine sarcoma. They comprised 21 patients with uterine leiomyosarcoma (21/45, 46.67%), 19 with endometrial stromal sarcoma (19/45, 42.22%), and five with uterine adenosarcoma (5/45, 11.11%).

### Surgical procedures and findings

Of the 6625 study patients, 1902 underwent laparoscopic surgery, 4449 trans abdominal surgery, and 274 hysteroscopic surgery. The incidence of unexpected uterine sarcoma was six of 1902 patients undergoing laparoscopic surgery, two of 274 undergoing hysteroscopic surgery, and 37 of 4449 patients undergoing transabdominal surgery; these differences are not statistically significant (*P* = 0.062), And 4060 patients underwent tumor resection and 2565 total hysterectomy. Sixteen of the patients who underwent myomectomy or tumor resection and 29 of those who underwent total hysterectomy were diagnosed as having unexpected uterine sarcoma. The data is shown in Table 1.

**Table 1:** Incidence of Unexpected Uterine Sarcoma in different Surgical approach in Tianjin of 6625 patients from 2011 to 2016

	Laparoscopic surgery	Transabdominal surgery	Hysteroscopic surgery	Tumor resection	Total hysterectomy	*P* value
Sarcomas (n)	6	36	3	14	31	0.062^※^
Fibroids (n)	1896	4413	271	4046	2534	< 0.001*
Total number (n)	1902	4449	274	4060	2565	
Incidence of UUS	0.32%^※^	0.81%^※^	1.09%^※^	0.34%*	1.2%*	

*P < 0.05 compared with the tumor resection and total hysterectomy groups

^※^*P* = 0.062 compared with the laparoscopic surgery, transabdominal surgery and hysteroscopic surgery groups

In the group of UUS, 11 of 14 patients who underwent rapid intraoperative frozen-section pathological examination were definitively diagnosed as having sarcoma on this basis, and two were diagnosed as having at least atypical leiomyoma (not excluding leiomyosarcoma, the diagnosis requiring confirmation by examination of paraffin-fixed sections) and one was diagnosed as having a uterine leiomyoma with severely atypical cells, plentiful nuclear divisions, necrosis, and invasive growth. Ten of the 11 patients underwent total hysterectomy and bilateral salpingo-oophorectomy and pelvic lymphadenectomy based on the results of the frozen-section examination. One of these patients underwent additional surgery 10 days after examination of paraffin-fixed sections. Six of the 34 patients whose diagnoses were not established intraoperatively underwent tumor resection only and refused reoperation, and the remaining 28 patients underwent additional surgery 8–45 days after the first operation.

### Clinical features of UUS and PDUS

#### Age and menopausal status

In the PDUS group, the ages of patients were between 24 and 68 years (mean age 50.21 ± 7.31). The ages of patients in the group of UUS were between 29 and 65 years (mean age 46.51 ± 8.14 years). One-way analysis of variance showed that the mean age differences between the two groups are statistically significant (*P* = 0.046), the UUS patients being significantly younger. In the PDUS group, 12 patients were pre-menopausal and 9 post-menopausal, whereas in the UUS group, 26 were pre-menopausal and 19 post-menopausal. The differences in menopausal status between the two groups are not statistically significant (*P* = 0.091). The data is shown in Table 2.

**Table 2:** Clinical features of unexpected uterine sarcoma and preoperative diagnoses of uterine sarcoma in Tianjin

	UUS (n = 45)	PDUS (n = 21)	*P* value
Age, years	46.51 ± 8.14	50.21 ± 7.31	0.046
Menopause, n (%)	19 (42.2%)	9 (42.8%)	0.961
History of disease, years	2.89 ± 1.79	3.52 ± 2.28	0.033
Clinical stage n (%)			< 0.001
I	37 (97.4%)	12 (70.7%)	
II + III	2 (2.6%)	9(29.3%)	
Primary complaints n (%)			0.319
Vaginal bleeding	23 (51.5%)	11 (51.2%)	
Vaginal discharge	5 (11.1%)	5 (23.8%)	
Abdominal pain	3 (6.7%)	2 (9.4%)	
Abdominal mass	7 (15.6%)	1 (4.8%)	
Compression	2 (4.4%)	1 (4.8%)	
Others	5 (11.1%)	1 (4.8%)	

Patients with unexpected uterine sarcoma were identified as UUS, and patients with preoperative diagnoses of uterine sarcoma were identified as PDUS

#### Patients’ chief complaints

In the UUS group, the commonest clinical manifestation was irregular vaginal bleeding (23/45, 51.1%), followed by abdominal mass (7/45, 15.6%), vaginal discharge (5/45, 11.1%), and abdominal pain (3/45, 6.7%), with symptoms of compression uncommon (2/45, 4.4%); (5/45, 11.1%) of the patients had no clinical manifestations, their uterine sarcomas being found incidentally during routine physical examination. In the PDUS group, the commonest clinical manifestations were irregular vaginal bleeding (11/21, 52.4%), followed by abnormal vaginal discharge (5/21, 23.8%), abdominal pain (2/21, 9.4%), abdominal mass (1/21, 4.8%), and compression symptoms (1/21, 4.8%); one patient was asymptomatic (1/21, 4.8%). There were no significant differences in clinical manifestations between the two groups (*P* = 0.594). The data is shown in Table 2.

#### Clinical staging

In the UUS group, there were 37 patients with Stage I, one with Stage II, and one with Stage III, whereas, in the PDUS group, 12 patients had Stage I, three Stage II, and six Stage III. PDUS group had more patients with Stage II and III sarcomas there is statistically significant (*P* = 0.0003). The data is shown in Table 2.

#### Duration of symptoms

In the PDUS group, the duration of symptoms was 3.52 ± 2.28 years, whereas in the UUS group the average duration of symptoms was 2.89 ± 1.79 years. The difference between the two groups is statistically significant (*P* = 0.033), the data is shown in Table 2.

### Prognosis

**Fig. 1:**
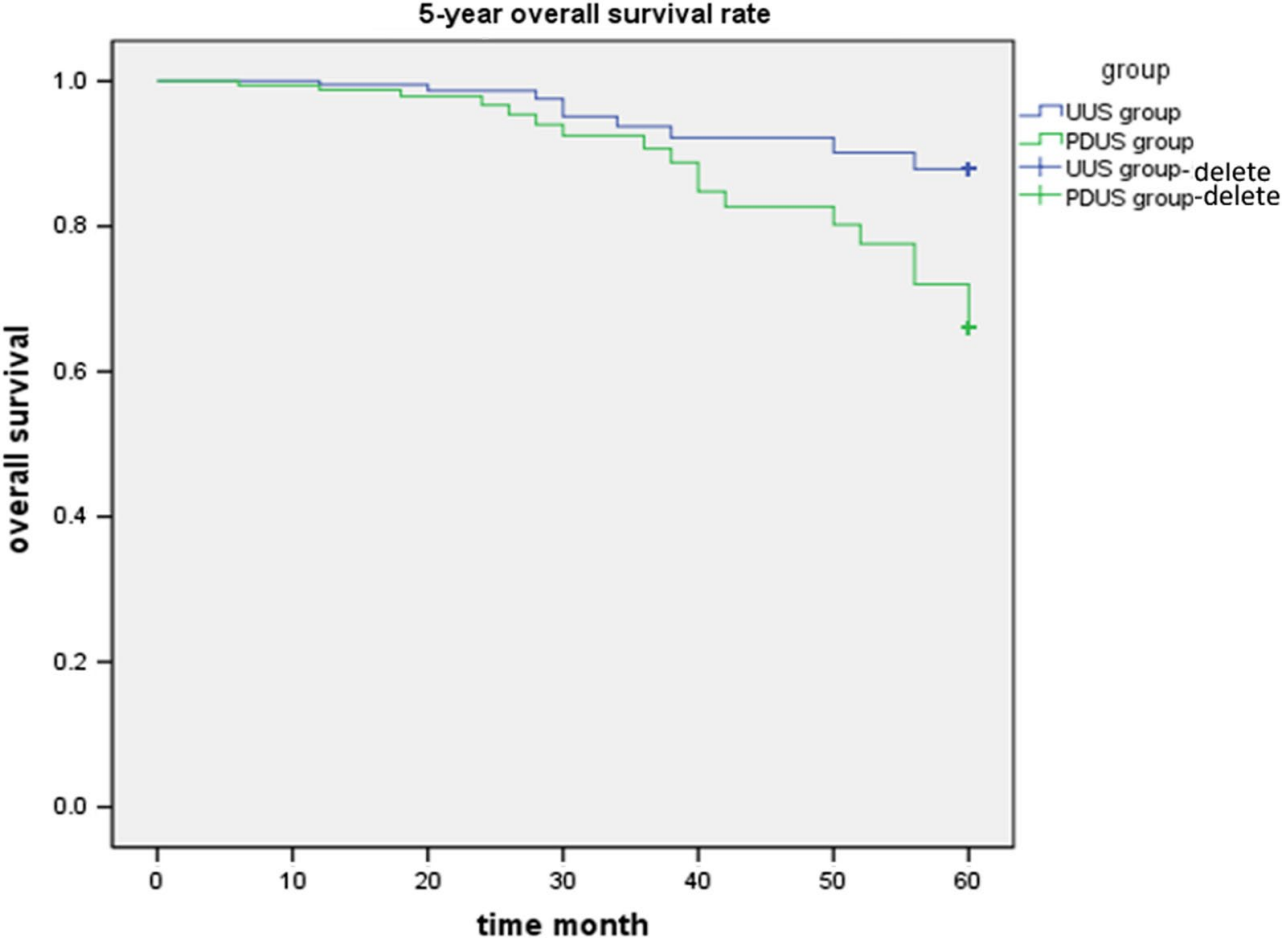
The Kaplan–Meier survival curve of the UUS and PDUS groups of patients in Tianjin

The all patients were followed up for 5 years, 10 cases died in the UUS group, and the 5-year overall survival (OS) rate was77.7%, in the PDUS group 9 cases died, and the 5-year survival rate was 46.3%. The average survival time of the UUS group and the PDUS group was 57.35 ± 0.17 months and 54.53 ± 0.25 months. There was a statistical difference between the two groups' OS rates (*X*^2^ = 292.645, *P* < 0.001), shown in Fig. 1. In the UUS group, two of the six patients managed by laparoscopic surgery underwent laparoscopic tumor resection using the morcellator. These patients subsequently underwent total hysterectomy and bilateral salpingo-oophorectomy 13–45 days after the first surgery; no abnormalities were found in the second surgery. The remaining four patients underwent laparoscopic total hysterectomy (LTH) in which the uterus was removed via the vagina without the use of a morcellator. Two of these patients underwent bilateral salpingo-oophorectomy in addition to LTH based on the results of the frozen-section examination; they were followed up for 60 months. No patient who had laparoscopic surgery underwent laparoscopic tumor resection using of morcellator and developed a recurrence.

## Discussion

Uterine fibroid is the most common benign tumor of the female. The prevalence of uterine fibroids in women aged less than 50 years is estimated at 70% [12]. Uterine sarcoma is rare and the UUS was only a part of uterine sarcoma. In this study, the incidence of unexpected uterine sarcoma was 0.67%, which is slightly higher than that previously reported (0.09–0.49%) [13, 14]. This discrepancy may be attributable to insufficiently large sample size or a lack of symptoms that would have alerted the attending physician to the correct diagnosis preoperatively. The difference in the incidence of unexpected uterine sarcoma between patients undergoing laparoscopic surgery and those undergoing trans-abdominal surgery was not statistically significant. However, the incidence of unexpected uterine sarcoma was higher in patients undergoing total hysterectomy than in those undergoing tumor resection, because UUS patients’ average age of onset is greater than 45 years, so were advised to undergo a total hysterectomy.

Common clinical manifestations of uterine sarcoma include irregular vaginal bleeding, lower abdominal mass, abnormal vaginal discharge, and compression symptoms. Because it is difficult to distinguish between these symptoms and those of uterine fibroids, the vast majority of patients with uterine sarcomas are preoperatively misdiagnosed as having uterine fibroids. Zhang et al. [10] reported a statistically significant difference in the incidence of compression symptoms between patients preoperatively diagnosed as having uterine sarcoma and those with unexpected uterine sarcoma (*P* = 0.018), maybe because patients with compression symptoms were eager to gain relief of uncomfortable symptoms through surgery and their preoperative evaluation was inadequate.

The advantages of laparoscopic surgery include reducing blood loss, time to wound healing, the incidence of wound infections, and duration of hospital stay, and enabling a quick recovery and return to daily activities. Because of these benefits, a growing number of patients, especially those with a single uterine fibroid or planning to undergo a total hysterectomy, prefer to undergo laparoscopic surgery. The literature suggested that the percentage of patients with uterine fibroids undergoing laparoscopic surgery reportedly increased from 11% in 2003 to 29% in 2013 [15]. Morcellators were first used in gynecological surgery in 1993 and were formally approved by the FDA for gynecological laparoscopic surgery in 1995; their use has been greatly promoted since then [16]. On 17 April 2014, the FDA issued a safety statement regarding the use of laparoscopic morcellator in uterine fibroid surgery [17]. The resected uterus should be removed per vagina or via an abdominal incision and resected fibroids should be removed after being crushed in a sealed specimen bag. Unexpected pathological diagnosis of uterine sarcoma after morcellation should be recorded and the patient closely followed up. However, the use of laparoscopic morcellation has also increased the risk of the spread of unexpected malignancies, especially uterine sarcoma. In recent years, some scholars have reported that patients with unexpected uterine sarcomas may have adverse surgical outcomes that are attributable to an increased risk of spread of malignancy associated with the use of electric uterine/fibroid morcellators during laparoscopic surgery. The risk of tumor is higher when an electric morcellator is used than in other laparoscopic procedures; however, the precise relative risk is not known [18]. It has been reported that the progression of disease was sped up in about 30% of the 53 unexpectedly discovered sarcomas discovered in 17,903 women undergoing surgery for fibroids and that 1/1000 morcellations will contribute to a poor prognosis [19]. Hinchcliff et al. have shown that the use of a morcellator increases the risk of abdominal/pelvic recurrence in patients (*P* = 0.001) and shortens the median tumor-free survival time (10.8 vs. 39.6 months; *P* = 0.002) but does not affect the overall survival [20]. Yang reported multiple pelvic recurrences in one patient 240 days after using a morcellator and considered that the use of the morcellator may have been in part responsible for the recurrences [21]. Hur et al. have shown that 0.28% of patients with uterine fibroids who have undergone total hysterectomy have unexpected uterine sarcoma and that in these patients, performing morcellation during laparoscopic total hysterectomy reduces the 5-year overall survival rate by 27% and shortens the recurrence-free survival time by 28.8 months [22]. Han et al. studied the rate of unintended morcellation of uterine smooth muscle tumors of uncertain malignant potential (STUMPs) was relatively high at 0.26% (10 in 3785), however, that for uterine sarcomas was 0.05% (2 in 3785) and the risks of unintended morcellation were very low for sarcomas and STUMPs [23]. Zhang et al. also reported the malignancy was identified in 5 (0.2%) of 3068 women who were treated by laparoscopy with power morcellation and 4 (0.3%) of 1180 who underwent laparotomy (*P* = 0.274), All nine patients were alive after a mean follow-up of 31.2 months in the laparoscopy group and 40.5 months in the laparotomy group [24]. Six of the 45 patients underwent laparoscopic surgery, four patients LTH, and two laparoscopic tumor resections; a morcellator was used in two of the patients who underwent laparoscopic tumor resection. No tumor spread or recurrence was found during the second surgery 2 weeks later or during follow-up. However, one patient who underwent a total hysterectomy had a recurrence 11 months after surgery. In this study, the use of laparoscopic morcellation did not increase the recurrence rate because we used the in-bag morcellation method. However, further larger studies with longer follow-ups are needed.

Patients with uterine fibroids generally have a long history with many outpatient checks, so changes in their fibroids should be documented in and records ultrasound reports rapid growth of uterine fibroids or continued growth after menopause should attract attention. For patients with irregular vaginal bleeding and abdominal pain preoperative diagnostic curettage or hysteroscopy should be performed. Patients suspected of having fibroid degeneration or with ultrasound indications of irregular or unclear blood flow should undergo pelvic nuclear magnetic resonance examination and those in whom a definite diagnosis is not established should undergo laparotomy.

The rapid frozen-section pathological examination should be performed intraoperatively on brittle masses or masses with indistinct boundaries: this is the last opportunity to diagnose an unexpected uterine sarcoma in time to perform an appropriate procedure and thus avoid a subsequent second surgical procedure. In the study, 14 patients underwent rapid frozen-section examination and 11 of those patients underwent a total hysterectomy, bilateral salpingo-oophorectomy, and pelvic lymphadenectomy based on the results of the frozen-section examination, thus obviating the need for a second surgical procedure or laparoscopic surgery.

There are several limitations in this study that should be taken into consideration. First, the non-randomized, retrospective nature may limit standards for diagnosis and evaluation. Second, conducted in a Specialist Hospital of Obstetrics and Gynecology this study included many patients with more severe clinical presentations, including larger palpable mass which may not be representative of the whole population and lead to selection bias. In addition, the follow-up time may appear to be insufficient. Third, owing to the small number of patients in the survival analysis, the clinical significance of the prognostic factors should be accepted with caution. Moreover, our data were from a single center and the combined analysis of UUS in our study may decrease statistical power to demonstrate differences in prognosis.

## Conclusion

The incidence of unexpected uterine sarcoma is low. UUS have a younger age of onset, shorter medical history, earlier clinical stage, and better prognosis. To improve the diagnosis rate of uterine sarcoma, preoperative screening methods for sarcoma should be developed. To reduce the risk of unintended tumor dissemination, patients with suspected cases should be provided adequate information about their treatment options, moreover, should be provided appropriate treatment options.

## Data Availability

All data generated or analyzed during this study are included in this published article.

## Abbreviations

UUS: Unexpected uterine sarcoma
PDUS: Preoperatively diagnosed uterine sarcoma
OS: Overall survival
LTH: Laparoscopic total hysterectomy
NCCN: National Comprehensive Cancer Network
